# Supplementary material for: Influence of Pleistocene climatic oscillations on the phylogeography and demographic history of endemic vulnerable trees (section Magnolia) of the Tropical Montane Cloud Forest in Mexico
Source: PeerJ. 2021 Sep 28;9:e12181. doi: 10.7717/peerj.12181 (PMC8485838; doi:10.7717/peerj.12181)

**Peer J**

**Supplemental Information**

**Influence of Pleistocene climatic oscillations on the phylogeography and demographic history of endemic vulnerable trees (section *Magnolia*) of the Tropical Montane Cloud Forest in Mexico**

Yessica Rico, M. Ángel León-Tapia, Marisol A. Zurita-Solís, Flor Rodríguez-Gómez, Suria Gisela Vásquez-Morales

**Table S2.** Pairwise F_ST_ comparisons between *Magnolia schiedeana, M. pedraaze* and *Magnolia schiedeana* pop. Oaxaca. Statistically significant values in bold.

|  | ***M. pedrazae*** | ***M. schiedeana*** |
| --- | --- | --- |
| ***M. schiedeana*** | **0.42** |  |
| ***M. schiedeana* pop. Oaxaca** | **0.57** | **0.665** |

**Fig S1.** BPEC analysis using four concatenated cpDNA regions in *M. pedrazae*, *M. schiedeana* and *M. schiedeana* pop. Oaxaca. Haplotype network showing the coalescent relationships among haplotypes and *K*= 3 clusters. The haplotype ID is the same number as in figure 2B. The color of each haplotype denotes its most likely cluster membership to three different lineages as seen in figure 2D.


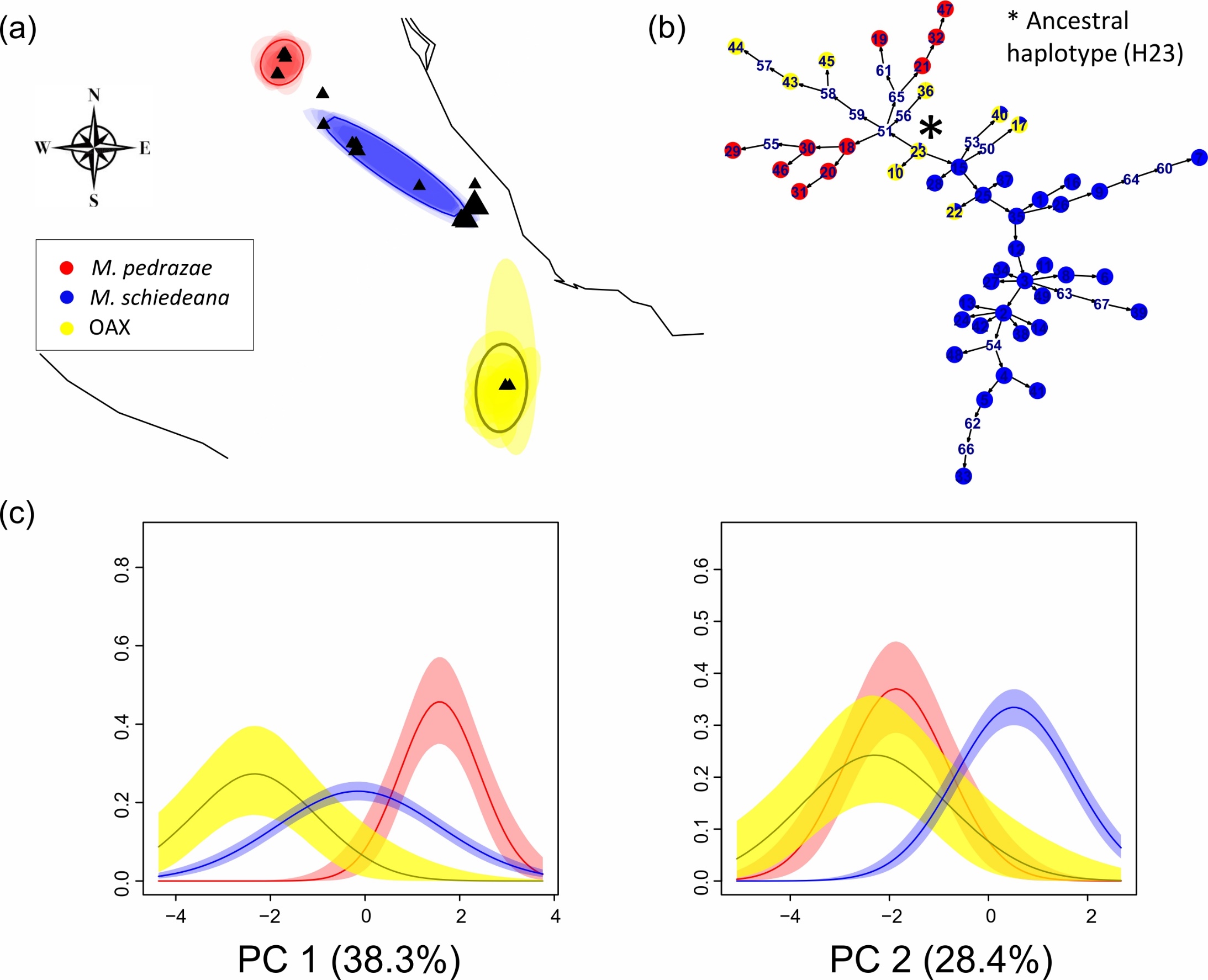


**Table S3.** Posterior parameter estimates for the scenario 2 (A) and scenario 5 (B) considering the three groups (*M.* *schiedeana, M. pedrazae, M.* *schiedeana* pop. Oaxaca). Estimates are based on 1% of simulated datasets closest to the observed values. Simulations and approximate Bayesian computation (ABC) analyses were performed considering sequence data. Parameters are *N* = effective population size for *M.* *schiedeana* (N1), *M. pedrazae* (N2), *M.* *schiedeana* var. Oaxaca (N3), and the ancestral populations (Na), *t* = time since divergence (generations), and *μ* = mutation rate for microsatellites.

(A)

| **Parameter** | **Median** | **2.50%** | **97.50%** |
| --- | --- | --- | --- |
| N1 | 9250 | 6900 | 9970 |
| N2 | 8280 | 3810 | 9930 |
| N3 | 6300 | 1340 | 9770 |
| NA | 5120 | 337 | 9710 |
| t1 | 3500 | 346 | 8130 |
| t2 | 8230 | 3770 | 9920 |
| µseq | 9.53^-8^ | 7.34^-8^ | 0.0000001 |
| Generations 10 yrs |  |  |  |
| t1 | 35000 | 3460 | 81300 |
| t2 | 82300 | 37700 | 99200 |

(B)

| **Parameter** | **Median** | **2.50%** | **97.50%** |
| --- | --- | --- | --- |
| N1 | 9360 | 7310 | 9970 |
| N2 | 8230 | 3580 | 9920 |
| N3 | 6010 | 1220 | 9760 |
| NA | 5420 | 413 | 9790 |
| t1 | 2290 | 173 | 7270 |
| t2 | 8480 | 4170 | 9940 |
| µseq | 9.49^-8^ | 7.35^-8^ | 0.0000001 |
| Generations 10 yrs |  |  |  |
| t1 | 22900 | 1730 | 72700 |
| t2 | 84800 | 41700 | 99400 |

**Fig S2.** Bayesian computation (ABC) analyses; model performance of the best performing scenario 2 and 5. (A) Posterior probability of scenarios assessed using a logistic regression on the 1% simulated data sets closest to the observed data. (B and C) model inspection process by performing a PCA on the test statistic vectors to visualize the fit between the observed and simulated data sets. Since the cloud of data from the prior (green) and observed (yellow) datasets are centred in the middle from the posterior predictive distributions, we concluded that the likely scenarios 2 (B) and 5 (C) explained well the observed data.


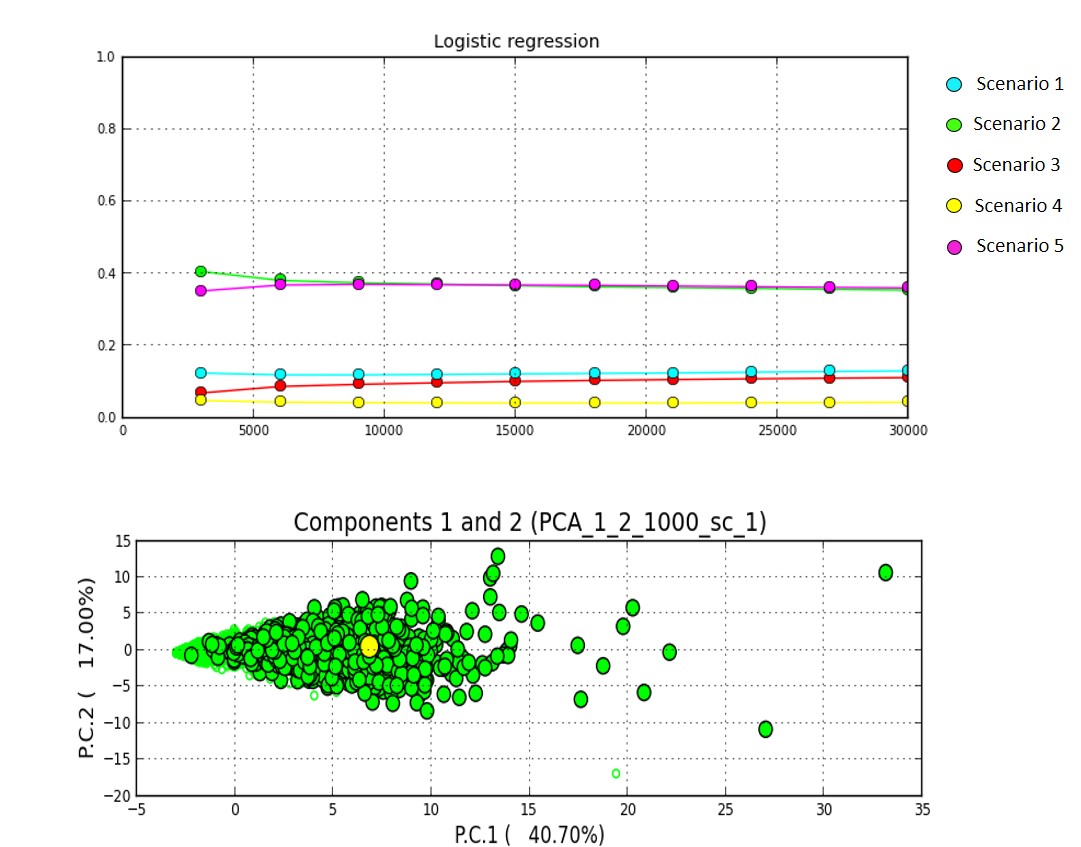


**(B)**

**(C)**


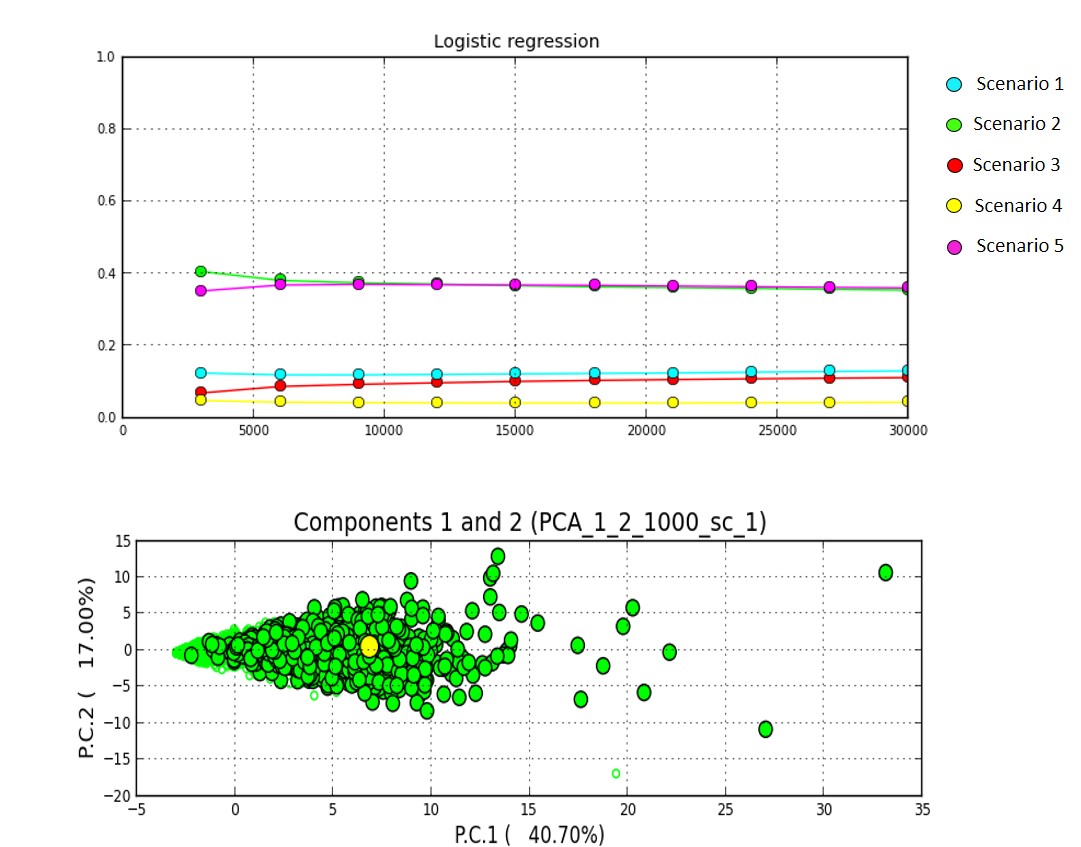


**(A)**

PC 2 (14.5%)

PC 1 (50.5%)

PC 1 (40.7%)

PC 2 (17%)

**Fig S3.** ExDet map showing the quantification of extrapolation due to covariate range (NT1) and correlation change (NT2) for the four past environments. For each past scenario, the NT1 (left; graded red) shows areas with at least one climate covariate outside the univariate range of reference data (current climate data), while NT2 (right; graded blue) shows areas that are within the univariate coverage of reference data, but non-analogous covariate combinations. In NT2 the areas ranging from 0 to 1 (green graded) are analogous to the reference data. The ExDet analysis was performed directly the similarity of pixels in the total area (calibration) between the reference (current climate) and projection (past scenarios) domains by accounting for both the deviation from the mean (NT1) and the correlation between variables (NT2)


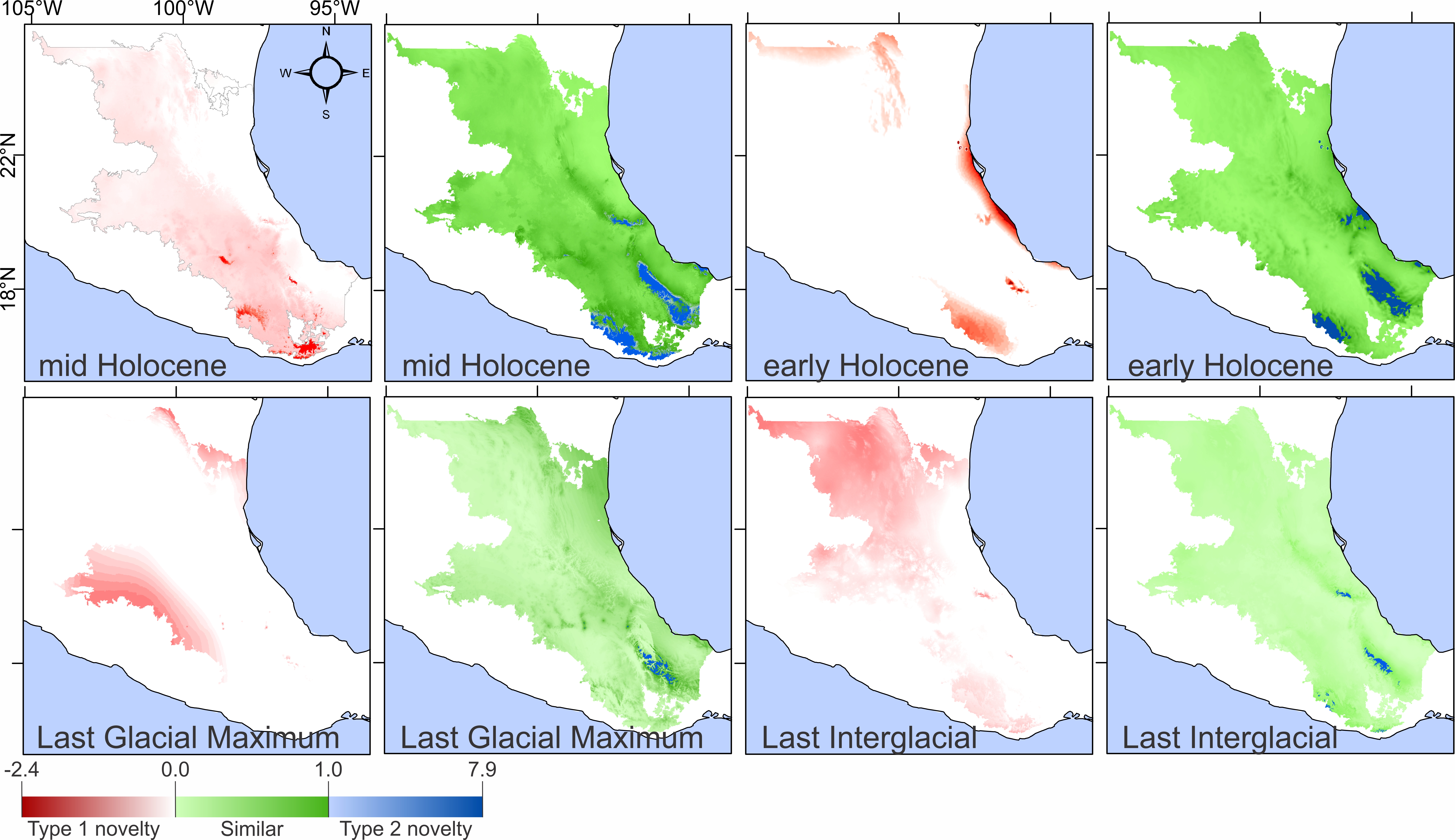

Supplement: Supplemental Information 2 [file peerj-09-12181-s002.docx]
